# Microfluidic Encapsulation Supports Stem Cell Viability, Proliferation, and Neuronal Differentiation

**DOI:** 10.1089/ten.tec.2017.0368

**Published:** 2019-03-15

**Authors:** Lorena Hidalgo San Jose, Phil Stephens, Bing Song, David Barrow

**Affiliations:** ^1^ Biomedical Engineering Research Group, Cardiff School of Engineering, Cardiff University, Cardiff, United Kingdom.; ^2^ Wound Biology Group, School of Dentistry, Cardiff Institute of Tissue Engineering and Repair, Cardiff University, Cardiff, United Kingdom.

**Keywords:** stem cells, biomaterials, microfluidics, cell encapsulation, neuronal differentiation, spinal cord injury

## Abstract

Stem cell encapsulation technology demonstrates much promise for the replacement of damaged tissue in several diseases, including spinal cord injury (SCI). The use of biocompatible microcapsules permits the control of stem cell fate *in situ* to facilitate the replacement of damaged/lost tissue. In this work, a novel customized microfluidic device was developed for the reproducible encapsulation of neural stem cells (NSCs) and dental pulp stem cells (DPSCs) within monodisperse, alginate-collagen microcapsules. Both cell types survived within the microcapsules for up to 21 days in culture. Stem cells demonstrated retention of their multipotency and neuronal differentiation properties upon selective release from the microcapsules, as demonstrated by high proliferation rates and the production of stem cell and neuronal lineage markers. When cell-laden microcapsules were transplanted into an organotypic SCI model, the microcapsules effectively retained the transplanted stem cells at the site of implantation. Implanted cells survived over a 10 day period in culture after transplantation and demonstrated commitment to a neural lineage. Our device provides a quick, effective, and aseptic method for the encapsulation of two different stem cell types (DPSCs and NSCs) within alginate-collagen microcapsules. Since stem cells were able to retain their viability and neural differentiation capacity within such microcapsules, this method provides a useful technique to study stem cell behavior within three-dimensional environments.

## Introduction

Most of the differentiated cells in adult tissues have a relatively short life span and are continuously replaced by new cells generated from stem/progenitor cells. In the adult mammalian organism, stem cells are found in almost all tissues and play a key role in maintaining cell genesis and renewal in different tissues and organs during the life span of the animal as part of the natural aging process, or after cell loss due to injury or disease.^1^

One of the main differences between embryonic and adult stem cells is their ability to differentiate into different cell types; that is, their potency. While embryonic stem cells (ESCs) are pluripotent, adult stem cells are multipotent or unipotent, in that their differentiation potential is more restricted and depends on their tissue of origin.^2^ However, recent studies have proved that these tissue-specific stem cells are able, under suitable conditions, to “transdifferentiate” toward a wider range of cell types, regardless whether these cells derive from different germ layers.^3^ These observations open a new spectrum of possibilities for adult stem cells to be used in regenerative medicine.

Adult stem cells can be found in the majority of tissues within the organism. Among these tissues, the bone marrow is the most widely used source for the isolation of stem cells, which can be subsequently expanded *in vitro.*^4^ Other sources of adult stem cells include adipose tissue,^5^ skeletal muscle,^6^ and skin.^7^ In 2000, Gronthos *et al.* described a clonogenic population of cells within the dental pulp that demonstrated high proliferative potential and tissue regeneration capability.^8^ In 2004, Nosrat *et al.* reported that dental pulp stem cells (DPSCs) could acquire a neuronal-like morphology and neuronal protein expression profile *in vitro.*^9^ Furthermore, we have also demonstrated the potential of these cells to differentiate into neuronal-like cells, as part of a preliminary study investigating repair of spinal cord injury (SCI).^10^

The main advantage of DPSCs over other adult stem cells is that they can be isolated through relatively noninvasive procedures. Healthy adult teeth, especially wisdom teeth, are often extracted during orthodontic treatment, while deciduous teeth are naturally shed in childhood. Dental pulp can then be excavated and cryogenically preserved. DPSCs can subsequently be isolated from such tissue, following thawing, with no detrimental effects in viability, proliferation, or differentiation.^11^ This has led to the founding of tissue banks that serve as stores of patient-matched progenitor cell samples ready to be accessed if and when required in the future.^12^

Although stem cell therapy represents a promising technique in tissue repair and regenerative medicine, there are still several drawbacks that need to be addressed. In a similar manner to organ transplantation, stem cell therapy requires the use of immunosuppressants to maximize cell viability, survivability and hence, efficacy.^13^ Furthermore, it is difficult to control end-stage cell fate within an organism, where the integration of the transplanted cellular material is challenging and difficult to achieve. To overcome these problems, researches have attempted to immobilize stem cells within biocompatible materials, such as hydrogels.^14^ A derivation of this is the encapsulation of the cells in biomaterial-based microcapsules, which permits the control of cell migration, differentiation, and tissue integration.^15–17^ Thus, the microcapsules provide the cells with a three-dimensional (3D) extracellular matrix environment that allows for a better control of cell parameters.^18^ Among the polymers used for cell encapsulation, alginate is the most commonly used due to its biocompatibility and relatively mild gelling conditions.^19^ However, it has been reported that alginate does not promote cell adhesion, a process that plays a key role in survival and function of many cell types.^20^ Therefore, alginate can be combined with collagens, such as type I collagen.^21,22^

Encapsulating techniques have been improved over the last few years, especially using microfluidic devices, and can enable the production of monodisperse droplets. This accurate control of capsule size can allow for the precise allocation of cells per microcapsule, and hence clinical delivery dose.^18^ Most of the studies on cell encapsulation use droplet extrusion methods, where cells are mixed with the appropriate polymer and then extruded through a needle, forming droplets that are collected in a gelling bath.^23^ However, the use of microfluidics allows for the formation of highly monodisperse microbeads, which can be gelled *in situ*, thereby critically reducing their production times.^24^

We hypothesize that the ability to successfully encapsulate stem cells with neural potential, using microfluidic techniques, would enable the future transplantation of such cell-based constructs into the injured spinal cord soon after injury, minimizing scar formation and enhancing host-tissue integration. Therefore, we have individually encapsulated DPSCs and neural stem cells (NSCs) within an alginate-collagen scaffold, using a novel customized microfluidic device.

We have demonstrated that the viability of the cells within the scaffold was maintained over extended time periods and, upon release, they maintained their proliferative and neuronal differentiation potential. The transplantation of the encapsulated cells in an organotypic model of SCI permitted the retention of the cells at the implantation site, demonstrating the significant benefits of using biomaterials to direct cell location within the host. After 10 days *in situ*, cells expressed neural markers, showing that DPSCs maintain their neural potential within the alginate-collagen microcapsules in the *ex vivo* SCI model.

## Materials and Methods

### Animals

Twenty-one to 28 day old C57/Bl6 mice utilized for spinal cord tissue harvest were obtained from Charles River Laboratories, UK and maintained at the Joint Biological Services Unit at Cardiff University, Cardiff, UK. Mice were sacrificed by CO_2_ asphyxiation in accordance with Schedule 1 of the Animals (Scientific Procedures) Act 1986.

### Cell culture

#### Neural stem cells

NSCs were isolated from the cortex of E14 C57BL/6 mice as previously described.^25^ Cells were maintained in Dulbecco's modified Eagle's medium (DMEM)/Ham's F12 (1:1) containing 2.5 mM L-glutamine and 15 mM HEPES buffer (Life Technologies, UK) supplemented with 1% (v/v) penicillin/streptomycin, 2% (v/v) B27 supplement (Life Technologies), 20 ng/mL basic fibroblast growth factor (bFGF), 20 ng/mL epidermal growth factor (EGF) (both Peprotech, UK), and 10 μg/mL insulin-transferrin-sodium selenite supplement (ITSS) (Roche Life Science, UK). NSCs were cultured as floating neurospheres with half medium changes performed every 2 days. Neurospheres were subcultured every 6 days using accutase (Life Technologies) to digest aggregates into single cells.

#### Dental pulp stem cells

DPSCs were isolated from the incisors of 21–28 day old C57BL/6 mice as described by Young *et al.*^10^ Cells were cultured in α-modification Minimum Essential Medium containing 2 mM L-glutamine, ribonucleosides, and deoxyribonucleosides (Life Technologies). The medium was supplemented with 1% (v/v) penicillin/streptomycin, 20% (v/v) heat-inactivated fetal bovine serum (FBS) (Life Technologies), and 100 μM L-ascorbic acid 2-phosphate (Sigma-Aldrich, UK). Medium was changed every 2–3 days until cells reached 80–90% confluence. Upon reaching confluence, cells were dissociated by adding 0.25% (v/v) trypsin-EDTA (Sigma-Aldrich).

#### Encapsulation of stem cells within alginate-collagen microcapsules

**FIG. 1.**
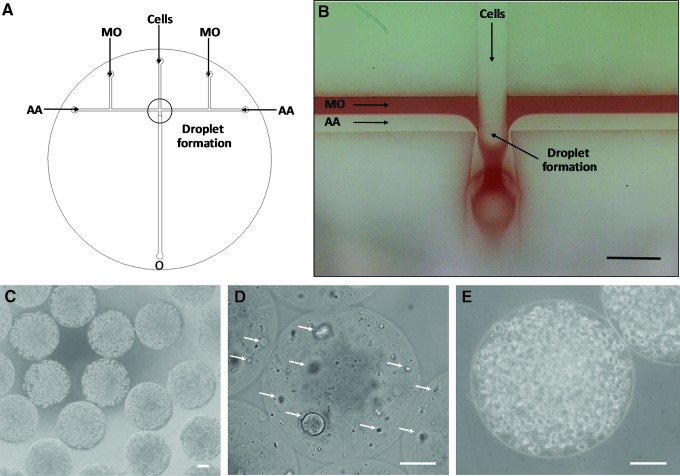
**(A)** Schematic representation of cell encapsulation within alginate-collagen microcapsules. The customized microfluidic device included one *middle* inlet for the introduction of the polymer solution containing the cell suspension. Microcapsules were produced by the shear force generated by the continuous phase formed by a laminar flow composed of MO and AA in mineral oil. **(B)** MO phase (*red*) acted as a shielding phase to prevent quick alginate gelation at the junction. Scale bar = 800 μm. **(C)** Highly monodisperse cell-laden alginate-collagen microcapsules of 440 ± 3 μm were produced with the customized microfluidic device. **(D)** Encapsulated NSCs growing in the form of neurospheres inside the alginate-collagen microcapsules (*white arrows*). **(E)** DPSCs were maintained as single cells within the microcapsules. Scale bars = 100 μm. AA, acetic acid; DPSC, dental pulp stem cell; MO, mineral oil; NSC, neural stem cell.

NSCs and DPSCs were individually resuspended in a polymer solution containing 2% (w/v) medium viscosity alginic acid (Sigma-Aldrich), 50 mM microcrystalline precipitated CaCO_3_ (Speciality Minerals, Birmingham, UK), and 2 mg/mL type I collagen (First Link, UK) at a density of 1 × 10^6^ and 1 × 10^7^ cells/mL, respectively. Cells were encapsulated within alginate-collagen microcapsules of 440 ± 3 μm diameter with a customized microfluidic device coupled with controllable syringe pumps (KD Scientific, UK) (Fig. 1A, B), for fluid delivery. Microcapsules were then collected in tubes containing prewarmed culture medium and, after washing with phosphate-buffered saline (PBS), they were suspended in fresh culture medium and transferred to the incubator (37°C, 5% CO_2_).

### Viability and proliferation

#### Trypan blue exclusion assay

Alginate-collagen microspheres were dissolved after a 5 min incubation at 37°C with 55 mM sodium citrate, followed by an additional 5 min incubation at 37°C with 1% (w/v) type I collagenase (both Sigma, UK), thereby releasing the cells. Cells were then centrifuged (100 *g* for NSCs and 400 *g* for DPSCs), supernatant removed, and resuspended in culture medium. Cell viability was estimated using trypan blue exclusion assay. Since NSCs grew in neurospheres, such aggregates were also digested by a 5 min incubation at 37°C with accutase before cell counting.

#### Live/dead assay and laser scanning confocal microscopy

Encapsulated cells were incubated with a solution containing 2 μM calcein and 2 μM ethidium homodimer-1 (EthD-1) (Life Technologies) in PBS for 30 min at 37°C. Subsequently, the distribution of live cells (green) and dead cells (red) was visualized using a Leica SP5 Confocal Microscope and Leica Application Suite Advanced Fluorescence (LAS AF) imaging software. Images of encapsulated cells were acquired from confocal Z scans over a depth of 400 μm. Acquired images were processed and overlapping images merged using freely available ImageJ software (https://imagej.nih.gov/ij/).

#### CellTrace™ Far Red staining for proliferation analysis by flow cytometry

DPSCs were stained with CellTrace Far Red Cell Proliferation Kit following the manufacturer's instructions (Life Technologies). Briefly, a stock solution of staining reagent was added to the cell suspension to give a concentration of 1 μM, and cells were incubated for 20 min at 37°C, in the dark. Culture medium containing 10% (v/v) FBS was added for 5 min to quench any free dye in solution. Cells were washed twice and seeded in flasks or encapsulated for further analysis using a FACSCanto flow cytometer (BD Biosciences, UK) coupled with HeNe 633 nm laser. Red fluorescence emission from CellTrace Far Red labeled cells (660/20 nm long pass filter) were measured. These data were then analyzed with FlowJo Version 10.2 software.

#### Inhibition of cell proliferation with mitomycin C

Negative controls of proliferation were prepared by treatment of DPSCs with mitomycin C according to manufacturer's instructions. Briefly, mitomycin C (Fisher Scientific, UK) was added to flasks containing 80–90% confluent DPSCs and culture medium to achieve a 10 μg/mL final concentration. Cells were then incubated for 3 h at 37°C in a humidified incubator with 5% CO_2_. Mitomycin C was removed and, after two washes with PBS, cells were trypsinized and seeded in flasks.

#### MTT assay

Cells were released from the microcapsules (see above), seeded in 96-well plates at a cell seeding density of 1000 cells/well, and allowed to settle down overnight. Twenty microliters of 5 mg/mL 3-(4,5-dimethylthiazol-2-yl)-2,5-diphenyltetrazolium bromide (MTT) solution (Sigma-Aldrich) were added to each well, and incubated for 4 h at 37°C/5% CO_2_. Culture medium was then removed and 150 μL of dimethyl sulfoxide was added to each well to dissolve any formed formazan crystals. Plates were incubated for an additional 30 min to allow the precipitate to dissolve completely. Absorbance was subsequently measured at a wavelength of 540 nm (FLUOstar Omega microplate reader; BMG Labtech). Before NSC seeding, plates were precoated (24 h) with 50 μg/mL poly-d-lysine (PDL) and 20 μg/mL laminin.

#### Apoptosis Tunel assay

Apoptotic nuclei in tissue sections were identified using the Click-iT^**®**^ Plus Tunel Assay (Thermo Fisher Scientific, UK) according to manufacturer's instructions. Briefly, tissue sections were incubated with 1× Proteinase K solution in PBS for 15 min. Samples were then incubated with EdUTP and TdT enzyme for 60 min at 37°C. This was removed and Alexa Fluor 594 dye containing copper protectant was then added to the tissues and incubated for 30 min at 37°C, protected from light. This solution was removed and after washing with PBS, tissues were mounted onto glass cover slips using mounting media supplemented with DAPI stain (VectorLabs, UK). Images of tissue sections were acquired with Deltavision imaging software with X/Y/Z multiple position recording (Applied Precision).

### Neuronal differentiation

#### NSCs neuronal differentiation

Encapsulated NSCs were released from the microcapsules 21 days after encapsulation and seeded at a density of 10,000 cells/cm^2^ on 50 μg/mL PDL/20 μg/mL laminin-coated plates in DMEM/F12 (1:1) containing 2 mM L-glutamine and HEPES buffer, 1% (v/v) penicillin/streptomycin, 2% (v/v) B27 supplement, 20 ng/mL bFGF, 20 ng/mL EGF, and 10 μg/mL ITSS. Neuronal differentiation was induced as previously described.^26^ Briefly, when cells reached 80–90% confluence, growth factors were gradually removed by half media changes every other day, for up to 10 days with growth factor-free culture medium.

#### DPSCs neuronal differentiation

Encapsulated DPSCs were released from the microcapsules 21 days after encapsulation and seeded at a density of 10,000 cells/cm^2^ on 10 μg/mL PDL/20 μg/mL laminin-coated plates. DPSCs were subjected to neuronal differentiation as previously described.^10^ Briefly, DPSCs were cultured in DMEM/F12 (1:1) containing L-glutamine and HEPES buffer, 1% (v/v) penicillin/streptomycin, 2% (v/v) B27 supplement, 20 ng/mL bFGF, 20 ng/mL EGF, and 10 μg/mL ITSS. After 5 days in culture, cells were washed with PBS and medium replaced with Neurobasal medium supplemented with 1% (v/v) penicillin/streptomycin, 2 mM L-glutamine, 1× non-essential amino acids (Sigma-Aldrich), 10 ng/mL nerve growth factor, 10 ng/mL brain derived neurotrophic factor, and 10 ng/mL neurotrophin-3 (all Peprotech).

#### Immunocytochemical staining

Cells and tissues were fixed with 4% (w/v) paraformaldehyde for 30 min at room temperature and then permeabilized in 0.1% (v/v) Triton X-100 for 30 min at room temperature. Nonspecific antibody binding was blocked by incubating in 5% (w/v) bovine serum albumin for 30 min. Samples were incubated overnight with the following primary antibodies: Nestin (10 μg/mL; Sigma), Sox2, Oct4 (10 and 2.5 μg/mL, respectively; Abcam, UK), β-III tubulin, Map2 (both 5 μg/mL; Cell Signalling, UK), and GFAP (2 μg/mL; Life Technologies). On the following day, complementary Alexa Fluor 488- and 594-conjugated secondary antibodies (both 4 μg/mL; Life Technologies) were applied. Glass coverslips were mounted using mounting media supplemented with DAPI stain (VectorLabs) and preparations imaged with Deltavision imaging software with X/Y/Z multiple position recording (Applied Precision).

#### Dissection and preparation of murine spinal cord explants

Spinal cord slice cultures were prepared as previously described.^27^ Briefly, from the 21–28 day old C57BL/6 mice complete spinal cords were dissected on ice. Cords were then washed twice in PBS supplemented with 1% (v/v) penicillin/streptomycin. Cords were cut with surgical blades on ice into ∼1 cm long sections. These were transferred to the centers of 35 mm tissue culture dishes with the dorsal area facing upward. An injury was induced on the center of each section by removing part of the tissue with a scalpel. The resulting damage area was 2.5 mm long and 0.5 cm wide, approximately.

#### Transplantation of encapsulated cells in *ex vivo* SCI model

To allow for easy identification of transplanted cells, a mixed population of NSCs and fibronectin-adherent DPSCs were isolated as described before from transgenic mice expressing GFP. Three different cell types were encapsulated within alginate-collagen microcapsules: (1) undifferentiated DPSCs, (2) neuronal predifferentiated DPSCs, and (3) undifferentiated NSCs. Neuronal predifferentiated DPSCs were cultured in NSC growth medium for 5 days before encapsulation. Microcapsules were then transplanted with forceps into the *ex vivo* SCI model previously prepared. Six microcapsules were transplanted for each experimental condition and sealed in position with 30 μL of Matrigel^**®**^ (BD Biosciences). Spinal cords were cultured with DMEM/F12 medium containing 25 mM HEPES buffer supplemented with 1% (v/v) penicillin/streptomycin and 20% heat-inactivated FBS (all Life Technologies) for up to 10 days, with medium changes every other day.

#### Fluorescence quantification

Fluorescence quantification was performed with ImageJ software (https://imagej.nih.gov/ij/), utilizing Huang's algorithm to stablish the threshold to define the regions of interest. Mean fluorescence intensity per GFP-expressing cell was calculated.

### Statistics

Data are represented as mean ± standard error of the mean (SEM). Statistical significance was determined by Student's *t-*test. *p* < 0.05 was considered statistically significant.

## Results

### Stem cells can be effectively encapsulated in alginate-collagen microspheres

The production of stem cell-laden alginate-collagen microcapsules was carried out utilizing a flow-focusing device fabricated on a polytetra-fluoroethylene disc (Fig. 1A, B). The customized microfluidic device included one middle inlet for the introduction of the polymer solution containing the cell suspension. Microcapsules were produced by the shear force generated by the continuous phase formed by a laminar flow composed of mineral oil and acetic acid in mineral oil. Uncontrolled alginate gelation was prevented by the mineral oil (Fig. 1B; red) acting as a shielding phase. Highly monodisperse cell-laden alginate-collagen microcapsules of 440 ± 3 μm (*n* = 30) were produced (Fig. 1C).

The two stem cell types encapsulated demonstrated different behaviors within the alginate-collagen microcapsules. NSCs formed aggregates within the microcapsules (Fig. 1D). Small cell aggregates were visible around 3 days after encapsulation, which increased in size along the culture period (21 days). However, DPSCs resided within the microcapsules as single cells (Fig. 1E). Few cells migrated outside microcapsules and attached on flask surface around 10 days after encapsulation (data not shown).

### Stem cells survive microfluidic encapsulation

**FIG. 2.**
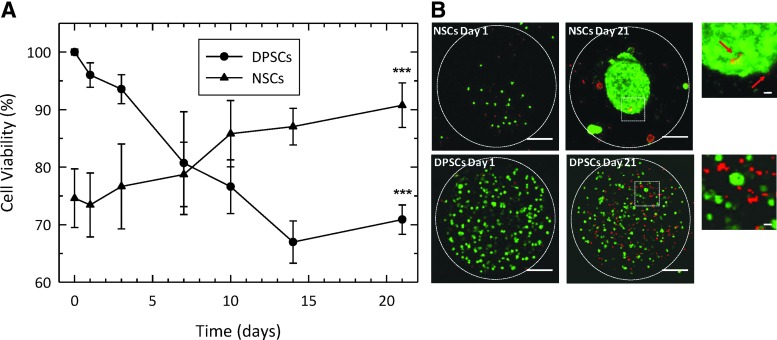
**(A)** Cell viability was estimated by trypan blue exclusion assay. The number of viable NSCs encapsulated within alginate-collagen microcapsules increased over the period tested (*triangles*). However, the viability of encapsulated DPSCs decreased after the 3 week period (*dots*). Data shown are mean ± SEM (*n* = 3) ***p < 0.001 (vs. first point). **(B)** Confocal images of encapsulated stem cells within alginate-collagen microspheres stained with calcein and EthD-1. Green fluorescence is emitted from live cells, whereas red fluorescence is emitted from dead cells. Increase in NSCs viability was confirmed by the formation of large, green cell aggregates within the microcapsules. The viability of encapsulated DPSCs decreased as observed by an increase in the number of red (dead) cells. Scale bars = 100 and 10 μm (*insets*). EthD, ethidium homodimer; SEM, standard error of the mean.

Stem cell viability within the capsules was assessed by trypan blue staining after the release of the cells from the beads (Fig. 2A). The viability of the encapsulated NSCs increased over the 21-day test period from 75% to around 90%. Conversely, a slight decrease in viability was demonstrated for the encapsulated DPSCs, although ∼70% of cells remained viable over the time course.

To confirm these findings *in situ*, encapsulated NSCs and DPSCs were stained with calcein and EthD-1 at day 1 and 21 postencapsulation (Fig. 2B). Confocal laser scanning microscopy images of the cells were acquired over a Z-scan depth of 400 μm. The appearance of both NSCs and DPSCs differed within the microcapsules. NSCs increased in number over the period of assessment and became aggregated into neurosphere-like structures. Furthermore, few dead cells (red) were observed.

For the DPSCs, however, cell number was relatively constant but the number of nonviable cells (red) increased, in agreement with the trypan blue viability results. Interestingly, throughout the time course of the investigation there was little evidence of the cells spreading out and attaching to the surrounding 3D scaffold within the bead. The random distribution of dead cells (red) for both cell types suggested an appropriate scale of the alginate-collagen microcapsules and that nutrient diffusion/waste build-up was not a limiting factor.

### DPSCs do not proliferate within alginate-collagen microcapsules

**FIG. 3.**
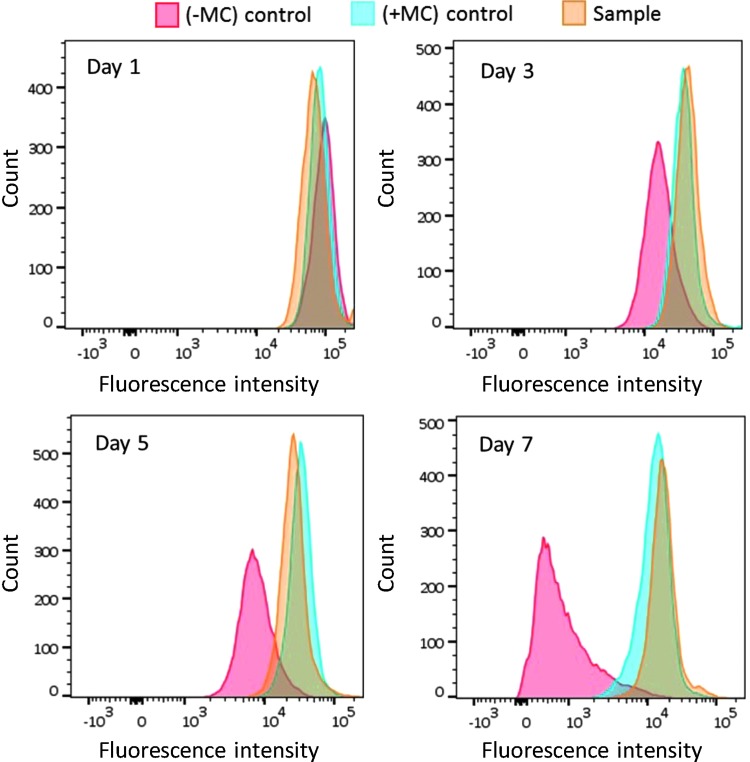
Study of DPSCs proliferation within alginate-collagen microcapsules. Both controls and samples were stained with CellTrace™ Far Red Cell Proliferation Kit and analyzed by flow cytometry over a period of 7 days. Mitomycin C treated cells (+MC) failed to divide as demonstrated by no decrease in the fluorescent signal. No mitomycin C treated cells (−MC) demonstrated cellular proliferation as evidenced by a shift and fall off of the fluorescent signal over a period of 7 days. No drop or shift in the fluorescence peak of encapsulated cells (sample) indicated that the DPSCs did not proliferate within the microcapsules.

NSCs proliferation within the microcapsules was confirmed by an increase in the number of viable cells by trypan blue exclusion assay and the observation of large cell aggregates by laser scanning confocal microscopy. However, the lack of proliferation by DPSCs required further confirmation. To this end, DPSCs were stained with CellTrace Far Red Cell Proliferation Kit and analyzed with flow cytometry. Before labeling, one group of cells was treated with mitomycin C to inhibit cell proliferation and then stained with the CellTrace Kit (+MC; negative control). A second group of cells was not treated with mitomycin C but labeled with the CellTrace Kit (−MC; positive control). The third group of cells was stained and encapsulated within alginate-collagen microcapsules (sample). Both controls (in form of cells monolayer) and encapsulated cells were cultured under standard conditions for up to 7 days and analyzed by flow cytometry (Fig. 3). Encapsulated cells were released from microcapsules before analysis.

Untreated monolayer cultures of cells (−MC; positive control) showed clear cellular proliferation as evidenced by a shift and fall off of the fluorescent signal as the cells divided over a period of 7 days and hence “shared” their fluorescent marker. Treated monolayer cultures of cells (+MC; negative control) failed to divide, as demonstrated by a consistent fluorescent signal. Prestained, encapsulated cells were released from the microcapsules (sample) after set periods of time in culture and analyzed. No drop or shift in the fluorescence peak indicated that the DPSCs did not proliferate within the microcapsules.

### Multipotency and neuronal differentiation potential are maintained after encapsulation

**FIG. 4.**
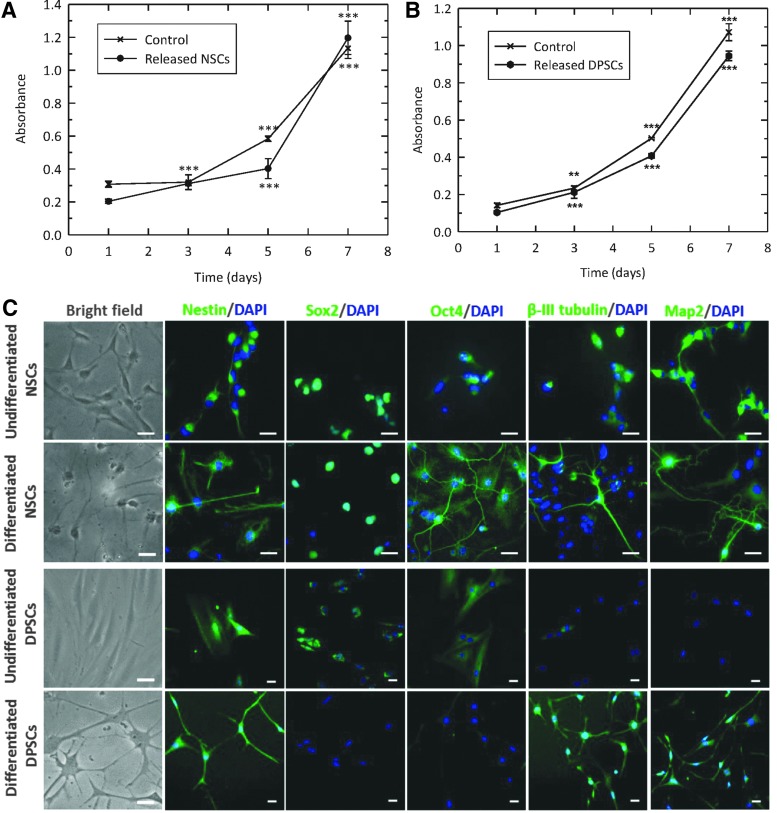
**(A, B)** NSC and DPSC proliferation upon release from alginate-collagen microcapsules. NSCs and DPSCs were released from microcapsules 21 days after encapsulation and seeded on 96-well plates. MTT proliferation assay was carried out on days 1, 3, 5, and 7 and absorbance measured at 540 nm. Both cell types (*dots*) showed similar growth rates to the nonencapsulated controls (crosses), suggesting that proliferation of cells was not compromised by encapsulation with alginate-collagen microcapsules. Data are shown as mean ± SEM (*n* = 3) **p < 0.01; ***p < 0.001 (compared to day 1). **(C)** Bright field and fluorescence images before and after neuronal differentiation of NSCs and DPSCs. Undifferentiated NSCs in growth medium show bipolar morphology with rounded bodies. Differentiated NSCs developed cell projections emerging from cell body, creating connections with adjacent cells. DPSCs before differentiation were typically bi-/tri-polar and fibroblast-like. After the differentiation protocol, DPSCs cell bodies adopted rounded morphology with multiple long processes sprouting out forming neuronal-like connections. NSCs and DPSCs were stained with antibodies against Nestin, Sox2, Oct4, β-III tubulin, and Map2, before and after differentiation. Scale bars = 25 μm.

NSCs and DPSCs were released from microcapsules 21 days after encapsulation and their proliferative potential assessed utilizing an MTT assay. Despite there being little evidence of proliferation, when the encapsulated DPSCs were released and then subsequently maintained in culture there was demonstrable proliferation for both DPSCs and NSCs (Fig. 4A, B). Indeed, while there were some subtle individual (time point) differences between encapsulated and control cells, the proliferative trend between these two cells types was very similar with distinct proliferation seen when each was compared with their respective day 1 value (*p* < 0.01 and *p* < 0.001).

With postencapsulated cells able to divide, their neuronal differentiation potential was next investigated. Encapsulated cells were liberated from the alginate-collagen microbeads 21 days after encapsulation and then exposed to a neuronal differentiation protocol. Undifferentiated NSCs demonstrated a bipolar morphology with rounded bodies (Fig. 4C). Upon differentiation, cells developed an increased number of extending processes and connected with adjacent cells. Undifferentiated DPSCs were typically bipolar and showed a fibroblastic-like appearance. After the differentiation protocol, cell bodies adopted a more rounded morphology with multiple long processes sprouting out forming neuronal-like connections.

Such a neuronal phenotype was investigated utilizing immunocytochemical analysis (Fig. 4C). Before and postdifferentiation, NSCs demonstrated evidence of both pluripotency markers (Sox2 and Oct4) and mature neuronal markers (β-III tubulin and Map2), suggesting a heterogeneous population of cells. The clear production of neuronal-like structures was evident after differentiation. Before differentiation, DPSCs appeared preneuronal with pluripotency markers (Sox2 and Oct4) switched on, and neuronal markers (β-III tubulin and Map2) switched off. However, postdifferentiation the opposite was demonstrated, indicating that during the 21-day encapsulation period, these stem cells retained their ability to differentiate toward a neuronal-like lineage.

### Encapsulated stem cells maintain functionality in an *ex vivo* spinal cord slice model

**FIG. 5.**
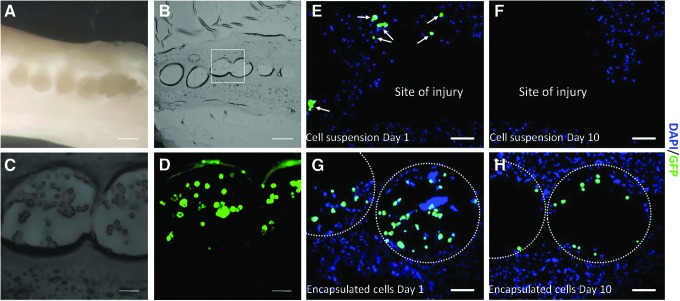
**(A)** Murine spinal cord was dissected after CO_2_ asphyxiation. An injury was induced on the dorsal part of the cord. Encapsulated cells were transplanted at the site of injury and sealed with Matrigel^**®**^. Scale bar = 500 μm. **(B)** Twenty micrometer thick frozen section of spinal cord. Scale bar = 500 μm. **(C, D)** Detailed images (*dotted square* in **B**) (bright field and fluorescence, respectively) showing encapsulated GFP DPSCs within the spinal cord. Scale bars = 100 μm. **(E)** Few unencapsulated cells could be observed within the tissue directly injected after implantation (*white arrows*). **(F)** Ten days postinjection, the injury site remained devoid of any nonencapsulated cells. **(G)** Encapsulated cells (*dotted circles*) were transplanted and visible at the injury site. **(H)** Encapsulated cells (*dotted circles*) were still visible 10 days after transplantation. Scale bars = 100 μm.

Six microcapsules containing an average of 2700 cells in total were transplanted into each *ex vivo* spinal cord slice culture (Fig. 5A). The addition of Matrigel on the top of the beads permitted the improvement of cell engraftment by sealing the microcapsules at the transplantation site, thereby avoiding the loss of beads during the culture period and preventing any potential spread of the injury. After Matrigel solidification, explants were incubated for fixed periods of time in culture then fixed, dehydrated, and cryosectioned into 20 μm thick slices (Fig. 5B–D). Alginate-collagen capsules containing GFP DPSCs could be observed in the middle of the cord, demonstrating the efficacy of Matrigel to retain the capsules at the site of implantation.

To highlight the benefits of the cell encapsulation technique for transplantation purposes, a comparison between the implantation of encapsulated and unencapsulated cells was carried out. The same number of cells corresponding to the transplantation of six beads loaded with cells (∼2700 cells) were pipetted into the site of injury, re-suspended in 2 μL of culture medium. Matrigel was then added on the top to seal the injury and to retain the cells at the site of implantation. Tissues were fixed, dehydrated, and sliced into 20 μm slices. Figure 5E and G demonstrate that cells could be observed with both methods of delivery immediately after transplantation. However, the longer-term localization of the cells differed according to the implantation method utilized. After 10 days, no GFP-positive cells were found at the injury site when cells were implanted as cell suspension (nonencapsulated) (Fig. 5F). Conversely, GFP-positive encapsulated cells were observed within the capsules at the injury site 10 days after transplantation (Fig. 5H).

**FIG. 6.**
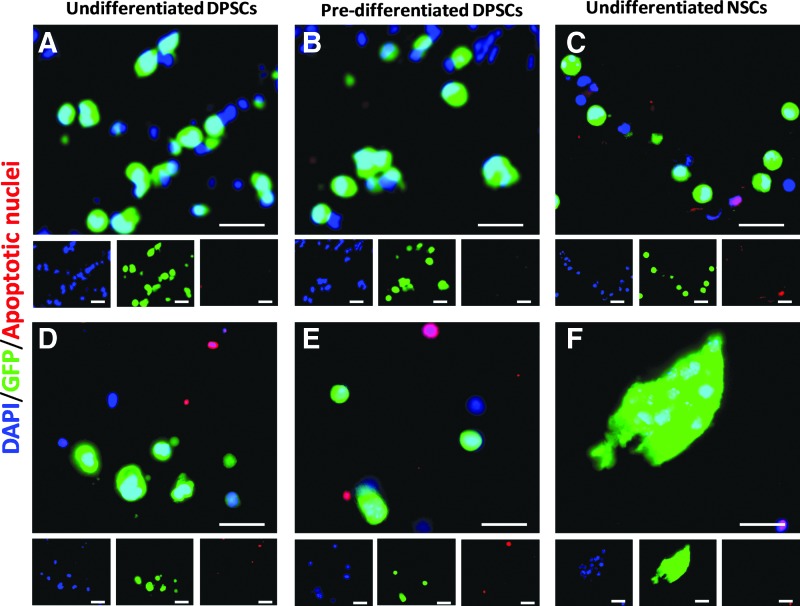
Apoptosis Tunel Assay of encapsulated cells transplanted into an *ex vivo* model of SCI. Co-expression of DAPI-stained nuclei (*blue*), GFP cells (*green*), and apoptotic nuclei (*red*). Encapsulated cells from the three conditions were transplanted and viability was studied using an Apoptosis Tunel Assay 0 **(A–C)** and 10 days **(D–F)** after transplantation. Condition 1: undifferentiated DPSCs **(A, D)**; condition 2: predifferentiated DPSCs into neuronal-like cells **(B, E)**; condition 3: undifferentiated NSCs **(C, F)**. Scale bars = 25 μm. SCI, spinal cord injury.

Undifferentiated DPSCs (cell type 1), predifferentiated DPSCs (cell type 2), and undifferentiated NSCs (cell type 3) were individually encapsulated and then transplanted into an organotypic model of SCI. Cell viability was studied both immediately (Day 0) and 10 days after transplantation. Cells were fixed, dehydrated, and cryosectioned before staining with Apoptosis Tunel Assay (Fig. 6). All three cell types were viable at the time of implantation, since no co-expression of DAPI/GFP/AF 594 was observed (Figure 6A–C). Ten days after transplantation, a number of apoptotic nuclei were observed for cell types 1 and 2, as observed by co-expression of DAPI/AF 594 (Figure 6D and E). However, some encapsulated cells were still viable for the three cell types. Furthermore, NSCs (cell type 3) proliferated within the tissue and started to form aggregates (Figure 6F). No signs of cell proliferation were observed for either DPSCs population. This behavior was similar to that observed in standard culture of encapsulated cells, where NSCs grew in the form of aggregates, but DPSCs did not proliferate.

**FIG. 7.**
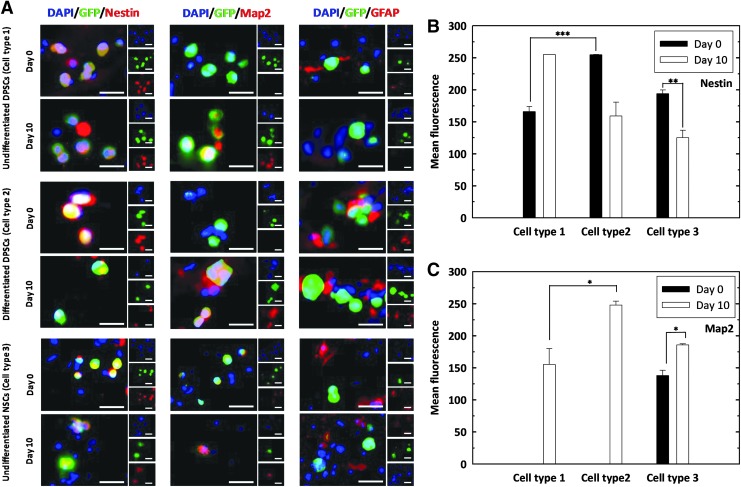
Expression of neuronal markers by transplanted cells from the three conditions. **(A)** Alginate-collagen microcapsules individually loaded with the three cell types were implanted in spinal cord slices and cultured for up to 10 days. Explants were stained with antibodies against Nestin, Map2, and GFAP before (day 0) and after culture (day 10). Scale bars = 25 μm. Fluorescence quantification of **(B)** Nestin and **(C)** Map2 markers. Data shown are mean ± SEM, *p < 0.05; **p < 0.01; ***p < 0.001.

All three cell types were encapsulated within alginate-collagen microcapsules, implanted into *ex vivo* spinal cord slices and cultured for up to 10 days. Tissue sections were fixed at days 0 and 10, dehydrated, and stained with antibodies against Nestin, Map2, and GFAP (Fig. 7). An intense endogenous staining of GFAP was observed in the spinal cord cultures, regardless of the cell type or the time point. However, none of the cell types investigated expressed GFAP at any time point.

Undifferentiated DPSCs (cell type 1) expressed the neural progenitor marker Nestin before and after culture within the section of the spinal cord. On the other hand, Map2, associated with more mature neuronal phenotypes, was not observed immediately after transplantation, but it was expressed after 10 days in culture.

For predifferentiated DPSCs (cell type 2) at day 0, Nestin expression was high and Map2 expression was not observed, which suggests that the predifferentiated DPSCs hold the neural stem/progenitor cells property at day 0. This Nestin expression was higher than that observed for undifferentiated cells at day 0, demonstrating that the predifferentiated DPSCs shows better neuronal differentiation capacity. However, predifferentiated DPSCs showed a sharply decreased Nestin expression at day 10, in contrast with an elevated Map2 expression at the same time, suggesting a dominant neuronal differentiation at this time point.

Unlike undifferentiated and predifferentiated DPSCs, undifferentiated NSCs (cell type 3) stained positively for Nestin and Map2 at day 0, being Nestin expression higher. This was reversed at day 10, where Map2 expression was increased.

## Discussion

The novel microfluidic method used in our lab permitted the production of highly monodisperse stem cell microcapsules, without the use of surfactants, and were gelled *in situ* in a matter of seconds. This rapidity is of considerable importance in terms of cell viability, since it permitted the quick transfer of the encapsulated cells into culture medium, thereby minimizing the risk of cell viability loss due to unfavorable conditions.

Cell encapsulation technology has been widely applied to improve grafts in tissue engineering applications, including bone and cartilage regeneration,^28^ skin wound repair,^29^ and treatment of dental defects.^30^ Immobilization of cells within hydrogels permits the accurate delivery of the cells at the injury site, allowing for a better performance of their therapeutic effect.^15–17^ In this article, we investigated the application of our customized microfluidic device for NSC and DPSC encapsulation, as part of our ongoing studies into SCI repair.

Cell viability studies demonstrated two different behaviors for the NSCs and DPSCs. The viability of NSCs increased over the investigation period, suggesting that cells proliferated within the alginate-collagen microcapsules. Laser scanning confocal microscopy showed an increase in the size of NSCs aggregates, supporting the results obtained with trypan blue exclusion assay.

Neurosphere growth in standard culture conditions occurs at a higher rate than that observed in encapsulated NSCs. Hence, it was deemed that encapsulation did not prevent proliferation of NSCs, but rather provoked a delay in cell growth. This delay in NSC proliferation permits maintenance of a higher cell viability than the conventional neurosphere culture method, where neurospheres tend to develop necrotic/apoptotic centers due to mass transport limitations.^31^ Similar studies have shown that nonencapsulated ESCs had a higher net growth rate when compared with encapsulated cells.^32^ Other investigations on the influence of alginate matrix on the proliferation of encapsulated NSCs demonstrated that their growth rate decreased with an increase in the hydrogel stiffness.^33^ Thus, proliferation of cells is affected by the mechanical resistance of the surrounding environment.

Unlike NSCs, DPSCs exhibited little sign of cell proliferation, indeed a decrease in the number of viable cells was observed. However, cell viability was still encouragingly high (∼70% of viable cells) up to 21 days after encapsulation. Interestingly, both bright field and confocal microscopy images of encapsulated cells showed little characteristic evidence of adhered cells, as they remained rounded within the alginate-collagen microspheres. Other studies on DPSCs immobilized within alginate hydrogels reported similar results.^34,35^ Kanafi *et al.* highlighted the different morphology of DPSCs depending on the culture conditions. When these cells were cultured in standard 2D culture they acquired fibroblast-like shapes, whereas when the cells were encapsulated within alginate microspheres they adopted rounded morphologies. The same behavior has also been observed for olfactory ensheathing cells, Schwann cells, and mesenchymal stem cells (MSCs) cultured on alginate hydrogels, where the cells acquired atypical spherical shapes and their metabolic activities were inhibited.^36,37^

DPSC lack of proliferation was further demonstrated by cell labeling with CellTrace Far Red Cell Proliferation Kit and analysis with flow cytometry. This finding is opposed to that reported by Kanafi *et al.*,^35^ where an increase in cell absorbance after addition of MTT reagent was observed over a period of 10 days in respect of DPSCs encapsulated within alginate beads. In the method developed by Kanafi *et al.*, the MTT reagent was directly added to plates where encapsulated cells were cultured. It is plausible that some of these cells might have escaped the beads, attached to the tissue culture surface of the plates and then proliferated (we have observed similar results for alginate only beads). However, the CellTrace method used in this work overcame the issue of potential cell escape (only viable cells directly isolated from the beads were analyzed) and demonstrated that DPSCs did not proliferate within alginate-collagen microcapsules.

Prolonged *in vitro* culture of viable, quiescent MSCs is well reported. MSC spheroid culture within collagen gels supports the maintenance of cell quiescence until regenerative stimuli were applied.^38^ Different studies have demonstrated that MSC spheroid culture prolongs replicative lifespan and delays cell senescence of MSCs *in vitro*,^39^ driving an increased expression of pluripotency marker genes (*Nanog*, *Sox2*, and *Oct4*).^40,41^ A different study on how to extend lifespan in yeast gives a hint of the potential mechanisms involved in the behavior of adherent stem cells encapsulated in low adherence hydrogels. Nagarajan *et al.* showed that encapsulated yeast cells within calcium alginate beads and fed *ad libitum* ceased to divide, but they maintained >95% viability over the course of 17 days. Analysis of gene expression of immobilized yeast cells demonstrated decreasing transcription of genes that regulate the cell cycle.^42^

A similar mechanism might take place in non-attached DPSCs within alginate-collagen microcapsules, where cells go into cell cycle arrest but continue to be metabolically active. Since cell aging is related to how many times a cell divides, controlled inhibition of cell division by encapsulation could allow for the temporal extension of stem cell lifespan. Hence, it was hypothesized that the alginate-collagen microcapsules produced here provide the cells with an artificial “niche” in which DPSCs reside in a quiescent state. This is like DPSCs residing within the dental pulp of living organisms, usually remaining quiescent when they are within the dental pulps, but responding quickly after injury.^43^

The maintenance of the multipotency and neuronal differentiation potential of cells after prolonged periods of time within microcapsules was investigated. Although the behavior of NSCs and DPSCs was altered under encapsulation conditions, upon release from the capsules, cells retained stem cell properties, as demonstrated by high growth rates and the expression of both stem cell and neuronal markers.

Both NSCs and DPSCs developed neuronal-like morphologies after application of a neuronal differentiation protocol previously developed in our group.^10^ Analysis of protein expression showed that NSCs expressed the neural markers Nestin, β-III tubulin, and Map2 and the stem cell markers Sox2 and Oct4 before and after differentiation. Co-expression of Nestin and β-III tubulin has been suggested to be involved in the formation of cell processes during the differentiation of NSCs.^44^ Although no variations in protein expression was reported by NSCs, it was observed that cells developed long axons and neurites emerging from cell bodies, both creating connections with adjacent cells and adopting a neuronal morphology.

Expression of Nestin by DPSCs was also observed both before and after differentiation. However, their behavior differed from that shown by NSCs in that DPSCs produced the stem cell markers Sox2 and Oct4 in the undifferentiated state but not after the differentiation protocol. In contrast, early stage neuronal markers β-III tubulin and Map2 were only visible in the differentiated state. DPSCs exhibited a change in their phenotype toward a neuronal-like morphology. Cells were typically bi-/tri-polar and fibroblast-like before differentiation. After the differentiation protocol, DPSC bodies adopted a rounded morphology with multiple long processes sprouting out forming neuronal-like connections. These results are similar to those reported previously in our group in nonencapsulated DPSCs,^10^ confirming that the encapsulation process did not affect DPSC capacity to undergo neuronal differentiation.

When encapsulated cells were transplanted into an *ex vivo* SCI model, it was demonstrated that microcapsules helped retain the grafted cells at the wound site, since no cells were found outside the implantation area. However, when cells were injected as cell suspension, no cells were found at the injury site after the culture period. The lack of extracellular matrix at the lesion site, which directs and organizes the wound healing cells, is one of the mechanisms that interferes with regenerative processes after SCI.^45^ Hence, stem cell encapsulation prior implantation into the damaged area facilitates the integration of transplanted cells.

The survival of the encapsulated cells after transplantation in the *ex vivo* model of SCI was studied after 10 days in culture. Encapsulated cells of three cell types survived throughout the culture period, as evidenced by limited apoptosis staining. Neither undifferentiated, nor neuronal predifferentiated DPSCs showed signs of proliferation, since no increased cellular density at the site of grafting could be observed. In contrast, NSCs showed some signs of cell proliferation, as indicated by the observation of small cell aggregates after the culture period. In relation to this, it has been demonstrated that immobilized neural progenitor cells within microfibers showed high proliferation rates after transplantation into an *in vivo* mouse model of SCI.^46^ Conversely, it has been reported that DPSCs do not proliferate after implantation into the mouse hippocampus but they stimulate proliferation of endogenous neural cells.^47^

The neural marker expression of encapsulated cells after transplantation was investigated using three different cell types: undifferentiated DPSCs, predifferentiated DPSCs, and undifferentiated NSCs. It is noteworthy to mention that none of the cell types investigated expressed GFAP at any time point. GFAP expression is associated with the formation of the glial scar after SCI, which has been regarded as inhibitory of axonal regrowth.^48,49^

At the time of implantation, undifferentiated and predifferentiated DPSCs showed distinct marker profiles. Nestin expression by predifferentiated DPSCs (cell type 2) was higher than for undifferentiated DPSCs (cell type 1). None of the DPSCs demonstrated positive staining for Map2. However, after 10 days in culture, both DPSC cellular populations stained positive for the neural markers Nestin and Map2. Expression of Map2 by undifferentiated cells and predifferentiated cells after the culture period suggests that the local environment provides signals that influence the fate of stem cells. Hence, transplanted DPSCs could have received signals from the spinal cord culture microenvironment that stimulated their differentiation toward neuronal lineages in the absence of external growth factors in the culture medium.

These results are comparable to those reported by Király *et al.*,^50^ where neuronally predifferentiated human DPSCs transplanted into the rat brain *in vivo*, expressed a number of neuronal specific markers, including N-tubulin, Neurofilament-M (NF-M), and NeuN. In a different study, when human DPSCs were transplanted into the avian embryo *in vivo*, DPSCs exhibited extended processes and demonstrated positive staining for both early and mature neuronal markers, β-III tubulin and NF-M, respectively.^51^

In contrast, NSCs stained positive for both Nestin and Map2 before and after culture, although differences in intensity were observed. The fluorescence intensity of these markers was reversed at day 10, being Map2 expression higher than Nestin. As mentioned previously, spinal cord cultures with transplanted NSCs showed the presence of cell aggregates after several days in culture, suggesting cell proliferation. As cells differentiate, their rate of proliferation usually decreases. Since NSCs were still proliferative within the tissue explants, and cells showed Nestin expression after the culture period, this suggests that these neural cells were not completely committed to the differentiation process.

The method described in this article provides the base for further development of more complex scaffolds for the treatment of SCI. Since this is a complex tissue and alignment of axons should be closely controlled, the scaffold needs to meet specific requirements for its successful use.
